# Whole Transcriptome Analysis Identifies the Taxonomic Status of a New Chinese Native Cattle Breed and Reveals Genes Related to Body Size

**DOI:** 10.3389/fgene.2020.562855

**Published:** 2020-11-03

**Authors:** Xiao-Dong Zheng, Jin Cheng, Wen-Juan Qin, Nyamsuren Balsai, Xuan-Jian Shang, Meng-Ting Zhang, Hong-Quan Chen

**Affiliations:** ^1^School of Animal Science and Technology, Anhui Agricultural University, Hefei, China; ^2^Key Laboratory of Anhui Local Livestock and Poultry Genetic Resources Conservation and Biobreeding, Hefei, China; ^3^Department of Dermatology, The First Affiliated Hospital of Anhui Medical University, Hefei, China; ^4^Key Laboratory of Dermatology (Anhui Medical University), Ministry of Education, Hefei, China; ^5^Key Laboratory of Major Autoimmune Diseases, Hefei, China; ^6^International Immunization Center, Anhui Agricultural University, Hefei, China

**Keywords:** whole transcriptome analysis, enrichment analysis, agricultural traits, taxonomic status, native cattle

## Abstract

Wandong (WD) cattle has recently been identified as a new Chinese native cattle breed by the National Commission for Livestock and Poultry Genetic Resources. The population size of this breed is less than 10,000. WD cattle and Dabieshan (DB) cattle are sympatric but are raised in different ecological environments, on mountains and plains, respectively, and the body sizes of these two breeds are markedly different. Blood samples were obtained from 8 adult female WD cattle and 7 adult female DB cattle (24 months old). The total RNA was extracted from leukocyte cells, and sequencing experiments were conducted on the Illumina HiSeq^TM^ 4000 platform. After the removal of one outlier sample from the WD cattle breed as determined by principal component analysis (PCA), phylogenetic and population structure analyses indicated that WD and DB cattle formed a distinct Central China cattle group and showed evidence of hybridization between *Bos. taurus* and *Bos. indicus*. The immune-regulator CD48 (*P* = 1.3E-6) was associated with breed-specific traits according to loss-of-function variant enrichment analysis. In addition, 113 differentially expressed genes were identified between the two breeds, many of which are associated with the regulation of body growth, which is the major difference between the two breeds. This study showed that WD cattle belong to the group of hybrids between *Bos. Taurus* and *Bos. indicus*, and one novel gene associated with breed traits and multiple differentially expressed genes between these two closely related breeds was identified. The results provide insights into the genetic mechanisms that underlie economically important traits, such as body size, in cattle.

## Introduction

As key providers of milk, meat, leather and labor, cattle are one of the most important livestock species worldwide and have been domesticated since the early Neolithic period (Loftus et al., 1994, 1999; Beja-Pereira et al., 2006). More than 1000 cattle breeds have been identified around the world (Leroy et al., 2016), and 57 indigenous cattle breeds have originated from and been domesticated in China, as described in the State Catalog of Livestock and Poultry Genetic Resource 2008. Extant domesticated cattle have been categorized by many researchers into two major geographic taxa: humpless taurine (*Bos. taurus*) and humped indicine (*Bos. indicus*) (Hiendleder et al., 2008; Gibbs et al., 2009; Canavez et al., 2012; Porto-Neto et al., 2013). Recent studies have indicated that cattle breeds from Central China show reliable evidence of having originated from hybridization between *Bos. taurus* and *Bos. indicus* (Lai et al., 2006; Lei et al., 2006; Mei et al., 2017; Chen et al., 2018).

Chinese cattle breeds vary in their intrinsic characteristics and are important genetic resources. However, there is pressure on the genetic diversity of cattle, and the worldwide extinction of some (local) cattle breeds is a major concern (Pariset et al., 2010; Biscarini et al., 2015). An interesting example is provided by the Wandong (WD) cattle breed, which was recently identified in Fengyang county, Anhui Province, by the National Commission for Livestock and Poultry Genetic Resources. WD cattle were domesticated in the watershed region between the Yangtze River and Huai River approximately 500 years ago and mainly exhibit two kinds of hair colors, yellow and brown. WD is one of the best breeds reared in China; it is characterized by a medium size and high-quality meat production that can reach the level of “3A plus” identified by the National Committee on Livestock and Poultry Genetic Resources. Additionally, it is one of the breeds that can be raised in the ecological environment in hilly areas with poor land and little rainfall. However, according to the statistics department data, the total breed population of WD cattle has declined alarmingly rapidly from more than 100,000 heads in the 1990s to less than 10,000 heads recently. Therefore, there is an urgent need to protect this precious genetic resource.

Dabieshan (DB) cattle were mainly domesticated in the eastern Dabie Mountains. These animals are small in size and suitable for farming in the mountains. Currently, the total breed population of DB cattle is more than 200,000 heads. The large population and purity of its genetic resources make DB cattle one of the best local livestock breeds for population genetic studies. Companies that raise cattle in this area mainly intend to maintain pure genetic resources. Therefore, it is rare for DB cattle to be in contact with other breeds. In the absence of gene flow, adaptive variants and linked variants through hitchhiking are expected to quickly increase in frequency within isolated populations (Rettelbach et al., 2019) and may evolve into differentiation islands through positive selection. In addition, genetic drift or purifying selection [background selection (BGS)] will reduce the population genomic diversity when a population is not large enough and will increase the number of differentiation islands between different populations (Burri et al., 2015). A gradual increase in the number of differentiation islands may have resulted in the evolution of this unique breed, which is able to adapt to mountainous environments.

In this study, we performed whole-genome RNA sequencing on these two phenotypically diverse domestic Chinese cattle breeds. By combining the obtained whole-genome RNA sequence data and whole-genome sequence data from 11 additional representative taurine and indicine breeds, we explored the taxonomic status of the WD cattle breed in more detail. Since loss-of-function variant association studies are considered extensions of genome wide association studies (Lu et al., 2017; Wen et al., 2018), we also investigated genes and corresponding variants that are associated with agriculturally important traits, such as size, through gene-level loss-of-function (LOF) variant enrichment analysis, which has proven to be efficient in detecting evidence of linkage in whole-genome sequencing data (Artomov et al., 2017; He et al., 2019; Yang et al., 2020). The aims of our analyses were to provide new insights into the population stratification of worldwide domestic cattle breeds and explore the relationship between genetic variants and agricultural traits.

## Materials and Methods

Animal experiments were executed according to the Institutional Animal Care and Use Committee (IACUC) guidelines under current approved protocols at Anhui Agricultural University.

### Sampling and Public Data Collection

**FIGURE 1:**
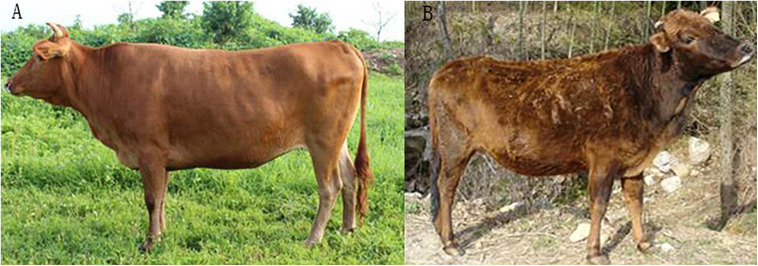
WD cattle and DB cattle. WD cattle **(A)** and DB cattle **(B)** were fed and maintained at the Daming Agriculture and Animal Technology Development Co., Ltd., FengYang County, Anhui Province, and the Anhui Wanjia Modern Agriculture Co., Ltd., Taihu County, Anhui Province, respectively.

Blood samples were obtained from 8 adult female WD cattle and 7 adult female DB cattle (24 months old) (Figure 1). WD cattle were fed and maintained under the same management conditions at the Daming Agriculture and Animal Technology Development Co., Ltd., which is located in FengYang county (Anhui Province) and has been designated as a WD cattle genetic resource conservation farm, while the DB cattle came from another conservation farm and were fed and maintained at the Anhui Wanjia Modern Agriculture Co., Ltd., Taihu County, Anhui Province.

We collected whole-genome RNAseq data from Qinchuan cattle (QC, *n* = 4, GSE47653), one of the most common local cattle breeds. In addition, whole-genome sequence data from 8 local Iran cattle (PRJEB6119) and 25 local Uganda cattle (PRJEB7061) were downloaded from the European Variation Archive^1^ website (details in Supplementary Table 5).

### RNA Extraction and cDNA Library Construction

The total RNA was extracted from plasma leukocyte cells extracted from samples collected from the jugular vein of each individual using TRIzol reagent (Life Technologies, United States) according to the manufacturer’s instructions. A TruSeq RNA Sample Preparation Kit (Illumina, United States) was used to purify the RNA and synthesize first- and second-strand cDNA. The quality was monitored with a NanoDrop ND-1000 spectrophotometer. The concentration of each sample was more than 50 pg/L, the amount of total RNA was more than 400 pg, and the RNA integrity number (RIN) was higher than 7.0. Then, an Agilent Bioanalyzer 2100 (Agilent Technology, United States) was used to determine the library fragment size distribution and assess whether it was suitable for computational analysis.

### High-Throughput Sequencing and Variant Calling

Sequence experiments were conducted by the Beijing Genomics Institute (BGI, Shenzhen, China) using the Illumina HiSeq^TM^ 4000 platform. Single reads (50 bp) obtained from sequencing were checked by FastQC (v0.11.5)^2^, and adapter sequences were removed with Trimmomatic (V0.36) (Bolger et al., 2014). Genome index files were generated by STAR (Dobin et al., 2013) using the gene annotation information GTF file (UCD1.2.95) and the *Bos. taurus* genome fast file (UMD3.1/bosTau6) (Zimin et al., 2009). Then, whole-transcriptome alignment was performed to obtain all splicing sites the first time. Thereafter, we used the STAR in the two-pass mode to rebuild the genomic index to obtain better alignments around novel splice junctions, and the clean reads were realigned to the *Bos. taurus* genome with Picard to mark duplicates with the default parameters. Variant calling was performed according to the best practices using the GATK HaplotypeCaller module (Van der Auwera et al., 2013) with the following quality control parameters: QualByDepth (QD) set to less than 2.0 and FisherStrand (FS) set to more than 30. ANNOVAR (Wang et al., 2010) was used to annotate variants relative to RefSeq annotations (UMD3.1).

### Phylogenetic and Population Structure Analyses

We obtained the PLINK genotype data format files by using the PLINK 1.9 “–vcf” command with the “–a2-allele” parameter, which will force the VCF (variant call format, generated by GATK program) file reference alleles to be set to A2. After that, the program of “convert” will calculate the number of copies of reference alleles per marker per individual (1 means one copy of the reference allele, 2 means two copies of the reference allele, and 9 means missing data) according to the PLINK SNP file. Principal component analysis was carried out with the overlapping common variants between the local data and the obtained public dataset to identify outlier samples using the smartPCA program of the EIGENSOFT (Patterson et al., 2006) package. A phylogenetic tree was constructed by using the neighbor-joining method in the program PHYLIP v3.695^3^, and the distance matrices (1 minus the identity-by-state value) were calculated using PLINK 1.07 (Purcell et al., 2007). The population structure was further inferred using ADMIXTURE (Alexander et al., 2009) with the kinship (K) set from 2 to 7.

### Whole-Genome Association Study

To identify genetic differentiation between these two typical cattle breeds, we performed single-variant analysis using Fisher’s exact test in PLINK with all variants passing quality control and calculated the genomic inflation based on the *P*-value distribution by the median chi-squared statistic (Yang et al., 2011). Thereafter, we performed multiple-variant testing with selected variants annotated as LOF variants (such as frame shift insertions, frame shift deletions, no frame shift insertions, no frame shift deletions, non-synonymous SNVs, stop gains, stop losses). Genes with two or more LOF variants were preserved to perform gene-level enrichment analysis. Three independent gene-based analysis methods, the Burden test, the sequence kernel association test (SKAT), and the optimal sequencing kernel association test (SKATO), were applied using the R package SKAT program (Wu et al., 2010, 2011), and one additional chi-squared test was performed with an in-house script.

### Differential Gene Expression Analysis

Clean reads in the RNA-seq data were mapped to the *Bos. taurus* genome (UMD3.1) using STAR aligner with the TranscriptomeSAM option. The output BAM files were processed using RSEM (Li and Dewey, 2011) to investigate gene expression levels. An in-house script was used to generate an expression matrix with transcripts per kilobase of exon model per million mapped read (TPM) values, which were obtained from the RSEM output gene abundance results. Based on a normal distribution and using log reads count values, a generalized linear model implemented in the R package limma (V 3.37.4) was used to assess differentially expressed genes (DEGs) using the empirical Bayes-moderated t statistics method (eBays). Differentially expressed genes with raw *p*-values lower than 0.05 and log2-fold changes larger than 334 (a mean value of the absolute value of the log2-fold change plus 2 multiple standard deviations of the absolute value of the log2-fold change) were considered significant. The R package ggplot2 (V3.1.0) was used to construct the volcano plot. To speculate about the potential biological processes related to bovine agricultural traits in which DEGs are involved, we performed the analysis of Gene Ontology (GO) and Kyoto Encyclopedia of Genes and Genomes (KEGG) pathways with the R package clusterProfiler (Yu et al., 2012).

## Results

### Alignments and Variant Identification

Fifteen samples from two cattle breeds (WD and DB) were sequenced using RNA-seq technology, generating an average of 24,135,850 raw sequencing reads, from which 23,893,462 clean reads remained after the filtering of low quality reads, and 21,054,824 uniquely mapped reads were finally obtained, with an average genome mapping ratio of 87.23 (Supplementary Table 1). A total of 724,998 single-nucleotide polymorphisms (SNPs) and 44,883 small insertions and deletions (InDels) were detected. A total of 20,819 variants were defined as LOF variants. After the removal of multiallelic variants (MNPs-multiple nucleotide polymorphisms, InDels), 20,575 variants remained. Then, variants (SNPs or InDels) with >90% call rates where each variant was uniquely located in one particular gene were retained. Additionally, the retained genes should have no less than LOF variants. Finally, we obtained 12,670 variants located in 2,720 genes for the subsequent gene-level enrichment analysis.

### Population Genetic Structure

Whole-genome RNA-seq data from four pooled QC cattle were aligned with data from fifteen local cattle that were subjected to the variant calling procedure described above. More than one million variants were detected. Two additional datasets were obtained, whole-genome sequence data from nine breeds from Uganda (25 individuals; 29,133,488 variants) and one breed from Iran (8 individuals; 19,999,624 variants). A total of 425,728 autosomal variants from these three datasets and were used for the population structure analysis.

**FIGURE 2:**
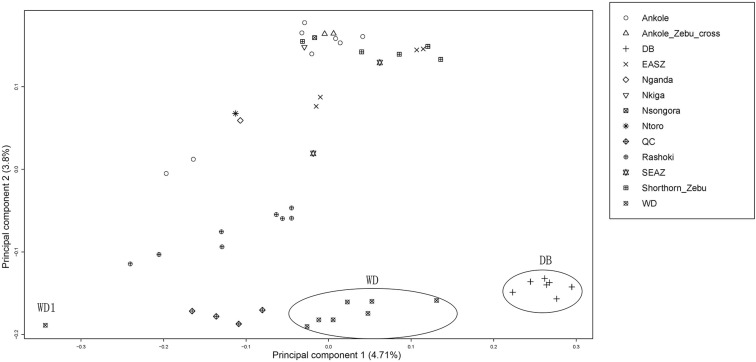
Principal component analysis of 11 cattle breeds. Principal component analysis (PCA) of 11 worldwide distributed cattle breeds.

**FIGURE 3:**
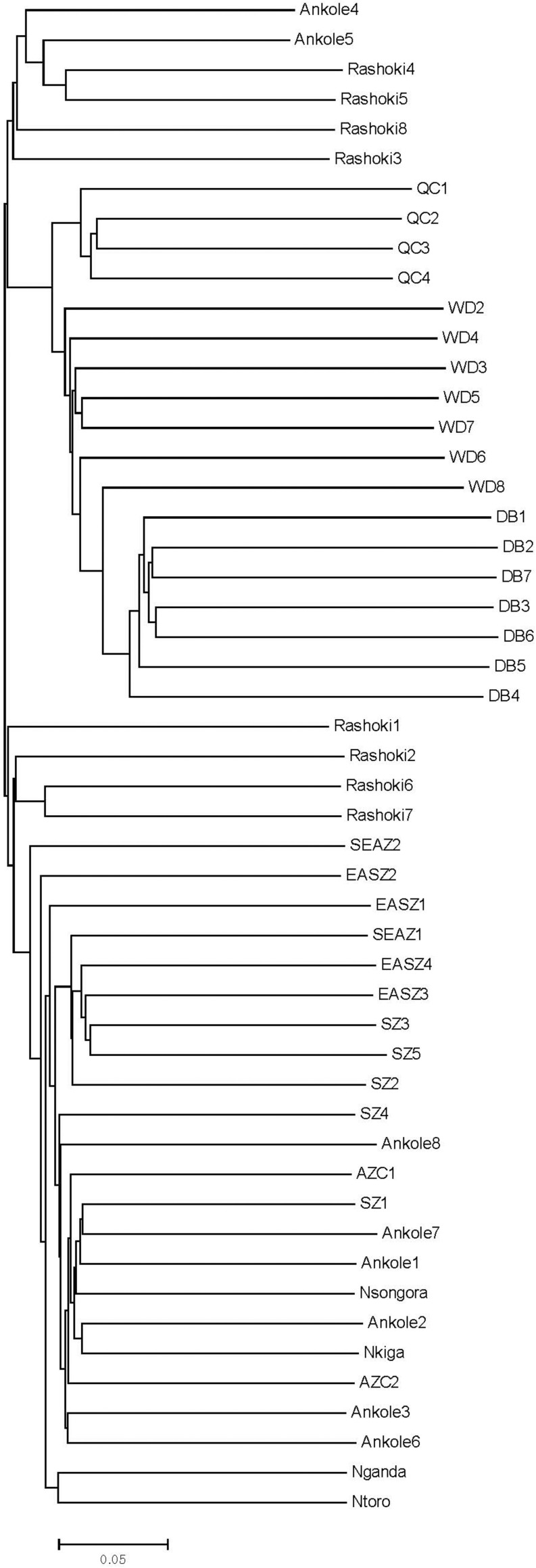
Phylogenetic tree of 11 cattle breeds. The neighbor-joining (NJ) phylogenetic tree constructed using whole-genome autosome SNP data.

**FIGURE 4:**
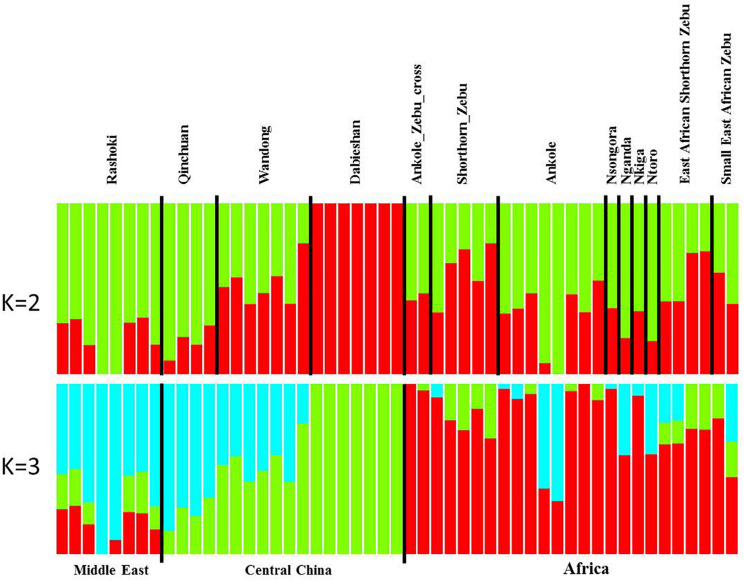
Genetic structure of 11 cattle breeds. Model-based clustering of cattle breeds using ADMIXTURE with *K* = 2 and *K* = 3. Breeds are arranged by geographic regions and labeled with the name of each breed.

Principal component analysis (PCA) demonstrated a clear genetic structure, with the samples from local Chinese cattle clustering together except for one sample, WD1, which was removed from the subsequent analysis (Figure 2). The same population affinities were recovered in phylogenetic trees constructed via the neighbor-joining (NJ) method (Figure 3). ADMIXTURE analysis also recapitulated these findings. To choose the best value for K, we set *K* = 2 to *K* = 7. When *K* = 2 or 3, we obtained the smallest cross-validation error (Supplementary Figure 1). Using these parameters we observed three geographically distributed ancestral components, labeled Middle East, Central China and Africa components (Figure 4). We noted that the QC, WD and DB breeds from three separate geographical regions formed a distinct Central China group. All cattle breeds from other regions (the Middle East and Africa) showed evidence of hybridization between *Bos. taurus* and *Bos. indicus*.

### Single Variant Association

Seven of the DB and seven of the WD cattle (one outlying sample was removed on the basis of PCA) were adult females (2 years old). It was obvious that the body size was significantly different between the two breeds (Table 1). To investigate the genetic linkage of this agricultural trait, we used 751,939 autosomal variants obtained from whole-genome RNA-seq of the two breeds, after performing quality control by excluding SNPs with a call rate of <90%, a minor allele frequency (MAF) <0.01, and Hardy–Weinberg equilibrium (HWE) in both cohorts <5e-8. The remaining 318,568 variants were employed to perform the whole-genome association analysis, and the distribution of –log10 *P*-values across all *B*. *taurus* autosomes used for the agricultural trait association of WD and DB cattle are shown in Supplementary Figure 2. After correction for the genomic inflation factor (λ_*gc*_ = 2.2742), there was no single variant showing evidence of a whole-genome significant association.

**TABLE 1:** Agricultural traits of two typical cattle breeds.

Agricultural traits	breed	N	Min	Max	Mean	SD	95% CI
Height at withers (cm)	WD	7	124.7	130.4	127.17	2.2	126.34–128
Body length (cm)	WD	7	140.7	151.6	146.03	4.02	144.51–147.55
Bust size (cm)	WD	7	170.5	179.3	174.26	3.32	173.01–175.51
Weight (kg)	WD	7	379.1	437.2	410.2	23.67	401.25–419.15
Height at withers (cm)	DB	7	110.6	119.3	115.31	3.31	114.06–116.56
Body length (cm)	DB	7	122.9	129.5	125.87	2.11	125.07–126.67
Bust size (cm)	DB	7	153.2	160.2	156.39	2.39	155.49–157.29
Weight (kg)	DB	7	280.6	293.7	284.87	4.43	283.2–286.54

*N, number of cattle; SD, standard deviation; CI, confidence interval.*

### Statistical Tests of the Robust Genes

We performed multiple-variant testing with the rare variants through SKAT analysis in R. Although the quantile-quantile plot of SKAT, SKATO and Burden *P*-values suggested that there was only moderate genomic inflation or systematic bias in this study (Supplementary Figure 3), no gene could pass the Bonferroni correction *P*-value threshold (*P* < 1.84e-5, 0.05/2720). However, there were 87 genes with gene-based association *P*-values (P_skat, P_skato, and P_burden) of less than 0.05.

**FIGURE 5:**
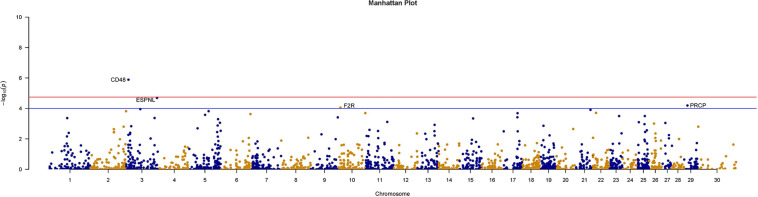
Manhattan plots of size-associated genomic variants based on the chi-squared test. Manhattan plots of -log10 (*p-*values) for two breeds based on the gene level LOF variant count chi-squared test method. The red horizontal line indicates the study-wide significance level following the Bonferroni correction for the gene-based method [-log10 (0.05/2720)], and the blue horizontal line indicates the suggestive significance level.

We further counted the minor allele numbers of these genes to fill the cross tabulation and performed the chi-squared test. With no evidence of genomic inflation (λgc = 0.1172), we observed a study-wide significant association for the CD48 gene (P_Chi = 1.3E-6, OR = 0.049, 95% CI = 0.011–0.209) and obtained suggestive evidence for an additional three genes (P_Chi < 1e-4) (Figure 5). Finally, there were 30 genes with a *P*-value of less than 0.05 according to four types of gene-level enrichment analysis methods (Table 2).

**TABLE 2:** 51 genes with *P*-values of less than 0.05 according to four independent gene-level LOF variant enrichment analysis methods.

Gene	N.Marker	A1	A2	B1	B2	OR (95% CI)	P_Chi	P_skat	P_skato	P_burden
CD48	10	2	138	32	108	0.049 (0.011–0.209)	1.3E-06	0.029818	0.007941	0.007113
ESPNL	4	18	38	44	12	0.129 (0.055–0.302)	2.04E-05	0.013621	0.006	0.003257
PRCP	8	34	78	69	43	0.272 (0.156–0.473)	6.47E-05	0.024092	0.024114	0.024528
F2R	8	1	111	22	90	0.037 (0.005–0.279)	8.83E-05	0.004401	0.004521	0.000835
TCTN1	9	2	124	23	103	0.072 (0.017–0.314)	0.000207	0.041766	0.02924	0.026316
HPS1	13	19	163	49	133	0.316 (0.178–0.563)	0.000995	0.035728	0.004116	0.003033
VPS72	3	12	30	30	12	0.16 (0.062–0.412)	0.001485	0.021421	0.020794	0.020457
GPATCH3	2	16	12	28	0	Null	0.001598	0.01049	0.01049	0.01049
RPAP3	4	6	50	24	32	0.16 (0.059–0.434)	0.002042	0.046709	0.016822	0.017364
TTC27	7	2	96	18	80	0.093 (0.021–0.411)	0.002578	0.013399	0.013399	0.013399
TBL2	4	5	51	22	34	0.152 (0.052–0.439)	0.002767	0.041708	0.037379	0.036504
ALG12	8	28	84	55	57	0.345 (0.196–0.608)	0.002969	0.013395	0.013414	0.048094
NIF3L1	4	4	52	20	36	0.138 (0.044–0.439)	0.003543	0.007855	0.013035	0.012078
CLK1	2	3	25	16	12	0.09 (0.022–0.369)	0.003736	0.00129	0.002294	0.002294
LY86	5	0	70	12	58	Null	0.004374	0.01049	0.01049	0.01049
HSPA14	4	1	55	14	42	0.055 (0.007–0.431)	0.004617	0.029138	0.006702	0.006702
SMYD5	2	9	19	22	6	0.129 (0.039–0.43)	0.006692	0.036717	0.036866	0.038031
HCLS1	2	1	27	12	16	0.049 (0.006–0.416)	0.006978	0.006702	0.006702	0.006702
MAK16	3	3	39	16	26	0.125 (0.033–0.472)	0.00933	0.016705	0.004503	0.004503
TMEM242	3	9	33	24	18	0.205 (0.079–0.533)	0.010545	0.021164	0.025414	0.030723
NDUFB4	4	0	56	9	47	Null	0.020472	0.034965	0.01049	0.01049
TREM1	8	2	110	14	98	0.127 (0.028–0.574)	0.021371	0.041363	0.041363	0.020973
ARG2	2	0	28	8	20	Null	0.025172	0.034965	0.034965	0.034965
UMPS	3	3	39	14	28	0.154 (0.04–0.586)	0.030324	0.016628	0.007454	0.007454
SLC35A5	3	3	39	14	28	0.154 (0.04–0.586)	0.030324	0.011943	0.010195	0.010195
RPIA	3	0	42	8	34	Null	0.031466	0.01049	0.01049	0.01049
VAMP5	2	4	24	14	14	0.167 (0.046–0.607)	0.042298	0.037557	0.013045	0.013045
PRR5	4	2	54	12	44	0.136 (0.029–0.639)	0.042755	0.043415	0.031177	0.033217
ATP2A2	2	0	28	7	21	Null	0.046012	0.034965	0.034965	0.034965
TMEM19	2	0	28	7	21	Null	0.046012	0.034965	0.034965	0.034965

*N.Marker, the SNP number involved in genes; A1, minor allele count of WD cattle; A2, major allele count of WD cattle; B1, minor allele count of DB cattle; B2, major allele count of DB cattle; OR (95% CI), odds ratio value of the chi-squared test, 95% confidence interval; P_Chi, gene-level chi-squared test *P*-value; P_skat, gene-based test SKAT P value; P_skato, gene-based test SKATO *P*-value; P_burden, gene-based test Burden *P*-value.*

### Differential Gene Expression

Based on the eBays method, genes with associated *P*-values of less than 0.05 and fold changes greater than 334 were considered as significant. Compared with WD cattle, 86 genes that were upregulated and 27 genes that were downregulated in DB cattle showed significant changes in transcript abundance. After false discovery rate (FDR, Benjamini–Hochberg method) correction, 64 genes were observed to be upregulated, and 21 genes were observed to be downregulated (Supplementary Table 2). A total of 113 DEGs are included in the volcano plot (Supplementary Figure 4).

### Gene Ontology (GO) Annotation for DEGs and LOF Genes

In total, 151 GO terms were assigned to 130 DEGs, and 134 terms were significantly enriched (Benjamini–Hochberg adjusted *P* < 0.05). Among these terms, 99 corresponded to biological processes, 28 to cellular components, and 10 to molecular functions (Supplementary Table 3). There were 28 GO terms assigned to the 30 LOF genes, all of which corresponded to cellular components. Only one GO term (GO:0031094) exhibited a *P*-value of less than 0.05 (Supplementary Table 4) which was involved in the regulation of the platelet dense tubular network (GO:0031094, p_adjust = 9.56e-3). including one suggested F2R gene identified by LOF enrichment analysis.

## Discussion

The findings of this study confirmed and extended the results of previous studies (Mei et al., 2017; Chen et al., 2018). The observed phylogenetic and population structure indicates that these Central Chinese cattle breeds formed an exclusive genetic group. The PCA provided similar results, and PC2 tended to separate populations sampled in Africa and those from the Middle East and Central China. Consistent with the geographic distance, the combination of PC1 and PC2 places the Middle Eastern cattle breed Rashoki between the African and Central Chinese cattle breeds. These data show that WD and DB cattle breeds present extremely low heterozygosity (∼0.2 and 0.18, respectively), while the QC, Rashoki cattle and African cattle breeds all exhibit intermediate heterozygosity (∼0.34–0.38), similar to a previous study of African taurine and indicine breeds (Orozco-terWengel et al., 2015). In contrast to other Central Chinese cattle breeds, WD cattle and DB cattle are raised in isolated environments, on the plains between the Huai and Yangtze rivers and in the mountains, respectively. The relatively less gene flow in these two breeds results in lower heterozygosity and a higher inbreeding coefficient than in other Central Chinese breeds. Additionally, WD cattle recently faced an extinction crisis in which the herd was reduced to six thousand head in 2011.

It remains unclear how genetic changes gradually accumulated in the genome and how these diverging breeds evolved. Reduced gene flow may speed up speciation, leading to large regions around divergently selected trait loci that are protected from recombination between genomic regions containing different locally adapted alleles (Via and West, 2008). The speciation process of restricted recombination caused the retention of these locally adapted variants and linked neutral variants in the distinct population genomes via extensive hitchhiking, probably causing the original ancestor to evolve into these two breeds that are able to adapt to different environments.

The greatest differences in the physical phenotypes of these two breeds are their body size and adaptation to different environments. There are many genes that have been reported to be associated with the economic traits of cattle (Li et al., 2010; Pausch et al., 2017; Raza et al., 2018; Sun et al., 2018; Cheng et al., 2020). In particular, genes such as MYLK4 (Zheng et al., 2019), CRTC3 (Wu et al., 2018), LEPR (Guo et al., 2008), and LHX4 (Ren et al., 2010) have been reported to be associated with the growth traits of Chinese cattle breeds. As body size is one the most important economic/growth traits for the modern breeding industry, it is valuable to investigate the genetic dynamics of this trait.

In this study, variant association analysis indicated the existence of moderate genomic inflation between these two cattle breeds, and no associated SNPs were detected. Therefore, based on advantages in detecting associations such as higher efficiency (greater detection power, and lower criteria for multiple testing) (Chen and Wang, 2019), a gene-based association strategy was used to identify physical traits associated with specific genes. Three gene-based methods (SKAT, SKATO, and Burden) showed there was moderate genomic inflation and that no gene could pass the threshold of the Bonferroni correction *P*-value. However, the chi-squared tests showed no evidence of genomic inflation at the gene level (λ_*gc*_ = 0.1172), and a novel gene, CD48, was found to be significantly associated with the differences in the traits of these two breeds, even after Bonferroni correction. Additionally, three genes with a suggestive association were found: ESPNL (espin-like), PRCP (prolylcarboxypeptidase) and F2R (coagulation factor II thrombin receptor) (P_Chi < 1e-4).

The encoded CD48 protein is located on the surface of lymphocytes and other immune cells and participates in activation and differentiation pathways in these cells. PRCP encodes a member of the peptidase S28 family of serine exopeptidases, which can cleave C-terminal amino acids linked to proline in peptides such as angiotensin II and has been reported to be associated with leanness, cell proliferation and blood pressure regulation. One study showed that PRCP depletion reduces cell proliferation (Adams et al., 2013). F2R is an important member of the GPCR family that is highly expressed in osteoclasts. Another study demonstrated that F2r responds to RANKL (receptor activator of nuclear factor-κB ligand) stimulation to attenuate osteoclastogenesis by inhibiting both the F2r-Akt and F2r-NFκB signaling pathways, which leads to a reduction in the expression of osteoclast genes (Zhang et al., 2020).

Differential gene expression analysis identified 86 genes that were upregulated in DB cattle compared to WD cattle (Supplementary Table 2). Unexpectedly, more than 50 genes were related to the ribosomal unit and endoplasmic reticulum. Ribosomal proteins are essential for protein translation, and specific ribosomal proteins have been shown to play an essential role in tumorigenesis, immune signaling, and development. The targeted deletion of RPL29 (ribosomal protein L29) in mice showed that animals are viable but are up to 50% smaller than their control littermates at weaning age (Kirn-Safran et al., 2007). On the other hand, 27 genes were upregulated in WD cattle compared to DB cattle, most of which were related to immunoreactions, transcription factors, signal transduction and metabolism. Interestingly, one upregulated gene (TLN1, *P* = 8.13E-6) was involved in the spreading and migration of osteoclasts, which may be correlated with the LOF enrichment results. Among these genes, the transforming growth factor gene (TGFB1) was significantly upregulated in WD cattle (*P* = 8.65E-7). TGFB1 has been reported to be a key regulator of feed efficiency in beef cattle, and motif discovery analysis showed that most genes co-expressed with TGFB1 were enriched for binding sites related to master regulators of muscle differentiation (Alexandre et al., 2019). Furthermore, a large meta-analysis of human data including 46 genome-wide association (GWA) studies of 133,635 individuals, also highlighted a role of TGFB2 in the TGF-beta signaling pathway in regulating human height (Lango Allen et al., 2010). Searches in the Online Mendelian Inheritance in Man (OMIM) database by using the search keywords “short stature,” “overgrowth,” “skeletal dysplasia,” and “brachydactyly” revealed gene records of TGFBR1 and TGFBR2 (receptors of TGFB1 and TGFB2) and many ribosomal protein genes, consistent with our data.

## Conclusion

In summary, we performed a whole-transcriptome study for WD and DB cattle. By using publicly available global cattle data, phylogenetic and PCA analyses indicated that these Central Chinese cattle breeds formed exclusive genetic groups even within breeds of hybrid *Bos. taurus* × *Bos. indicus* cattle, and these results are consistent with the results of previous studies (Yu et al., 1999; Mei et al., 2017; Chen et al., 2018), which confirmed that these two local Chinese breeds originated from hybridization between *Bos. taurus* and *Bos. indicus*. LOF variant enrichment analysis identified one novel gene, CD48, and three genes with a suggestive association. Among these genes, F2R responds to RANKL stimulation to attenuate osteoclastogenesis, and the PRCP gene has been reported to be involved in cell growth and angiogenesis. Differential gene expression analysis showed that many ribosomal protein genes are upregulated in DB cattle, and many transcription factors and metabolism-related genes are upregulated in WD cattle. All of these genes are connected with the economic trait of body size. However, further large-scale studies are needed to understand the roles of these genes and to identify robust phenotype-associated genes/mutations with a large effect size. Our results indicate that whole-genome RNA_seq experiments are an efficient approach for investigating population genetic diversity and the genetic architecture of complex traits in mammals.

## Data Availability Statement

The datasets presented in this study can be found in online repositories. The names of the repository/repositories and accession number(s) can be found in the article/Supplementary Material.

## Ethics Statement

The animal study was reviewed and approved by the Anhui Agricultural University.

## Author Contributions

X-DZ carried out the data, statistical, and other analyses and wrote the original draft. JC carried out the original investigation and design of the project, sample collection, and project administration. W-JQ, NB, X-JS, and M-TZ carried out the investigation and formal analysis. H-QC carried out the conceptualization, funding acquisition, methodology, project administration, supervision, and writing, review, and editing of the manuscript with input from all other authors. All authors contributed to the article and approved the submitted version.

## Conflict of Interest

The authors declare that the research was conducted in the absence of any commercial or financial relationships that could be construed as a potential conflict of interest.

## Footnotes

1 https://www.ebi.ac.uk/eva/

2 http://www.bioinformatics.babraham.ac.uk/projects/fastqc/

3 http://evolution.genetics.washington.edu/phylip.html

## Supplementary Material

Supplementary Figure 1 ADMIXTURE cross-validation plot. ADMIXTURE cross-validation plot for the whole-genome autosome dataset of 11 worldwide cattle breeds set at *K* = 2 through 7.

Supplementary Figure 2 Manhattan plots of *P*-values based on the single-variant association test. Manhattan plots of -log10 (*p-*values) for two breeds based on the single-variant association test. The red horizontal line indicates the genome-wide significance level from Bonferroni correction [-log10 (5e-8)] for the single-marker analysis, and the blue horizontal line indicates the suggestive significance level [-log10 (1e-5)].

Supplementary Figure 3 Quantile-quantile plots of *P-*values. The observed negative logarithms of the *P-*values obtained using three types of gene-based methods for the two breeds are plotted against their expected values under the null hypothesis of no association.

Supplementary Figure 4 Volcano plot of DEGs for the two cattle breeds. DEG expression profiling [with transcripts per kilo-base of exon model per million mapped reads (TPM) values] was constructed for the two breeds. Red plots represent genes that are significantly upregulated, and green plots represent genes that are significantly downregulated.
