# Supplementary figures and images for: Whole Transcriptome Analysis Identifies the Taxonomic Status of a New Chinese Native Cattle Breed and Reveals Genes Related to Body Size
Source: Front Genet. 2020 Nov 3;11:562855. doi: 10.3389/fgene.2020.562855 (PMC7670488; doi:10.3389/fgene.2020.562855)

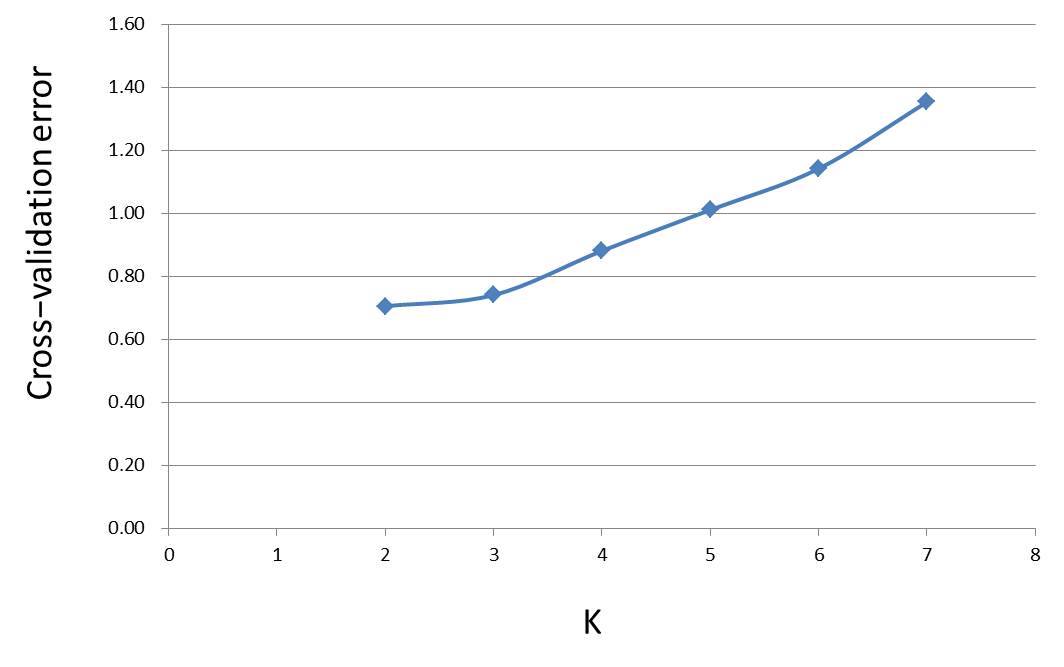

Supplement: Supplementary Figure 1 — ADMIXTURE cross-validation plot. ADMIXTURE cross-validation plot for the whole-genome autosome dataset of 11 worldwide cattle breeds set at K = 2 through 7. [file Figure_1.JPEG]

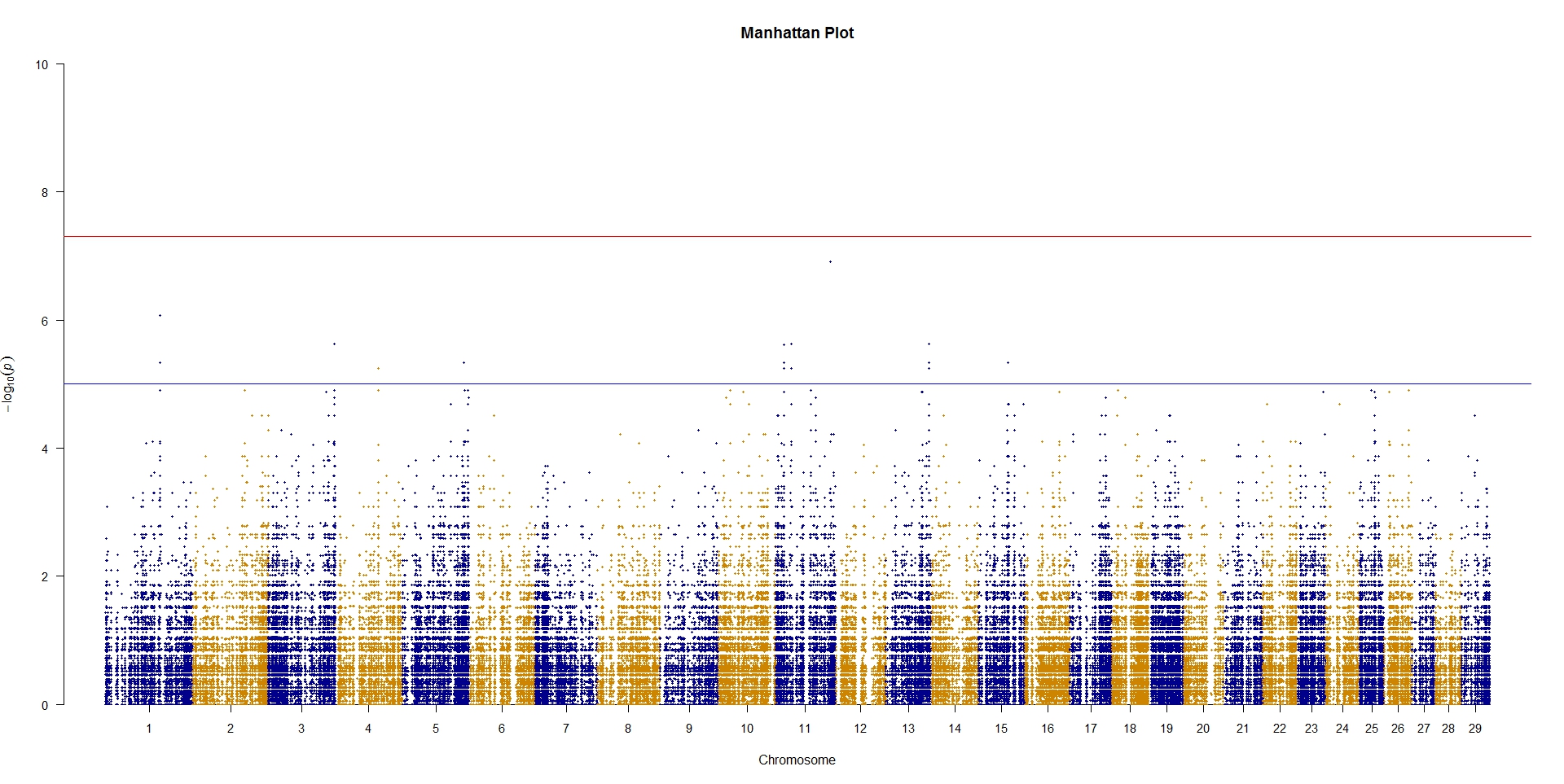

Supplement: Supplementary Figure 2 — Manhattan plots of P-values based on the single-variant association test. Manhattan plots of -log10 (p-values) for two breeds based on the single-variant association test. The red horizontal line indicates the genome-wide significance level from Bonferroni correction [-log10 (5e-8)] for the single-marker analysis, and the blue horizontal line indicates the suggestive significance level [-log10 (1e-5)]. [file Figure_2.JPEG]

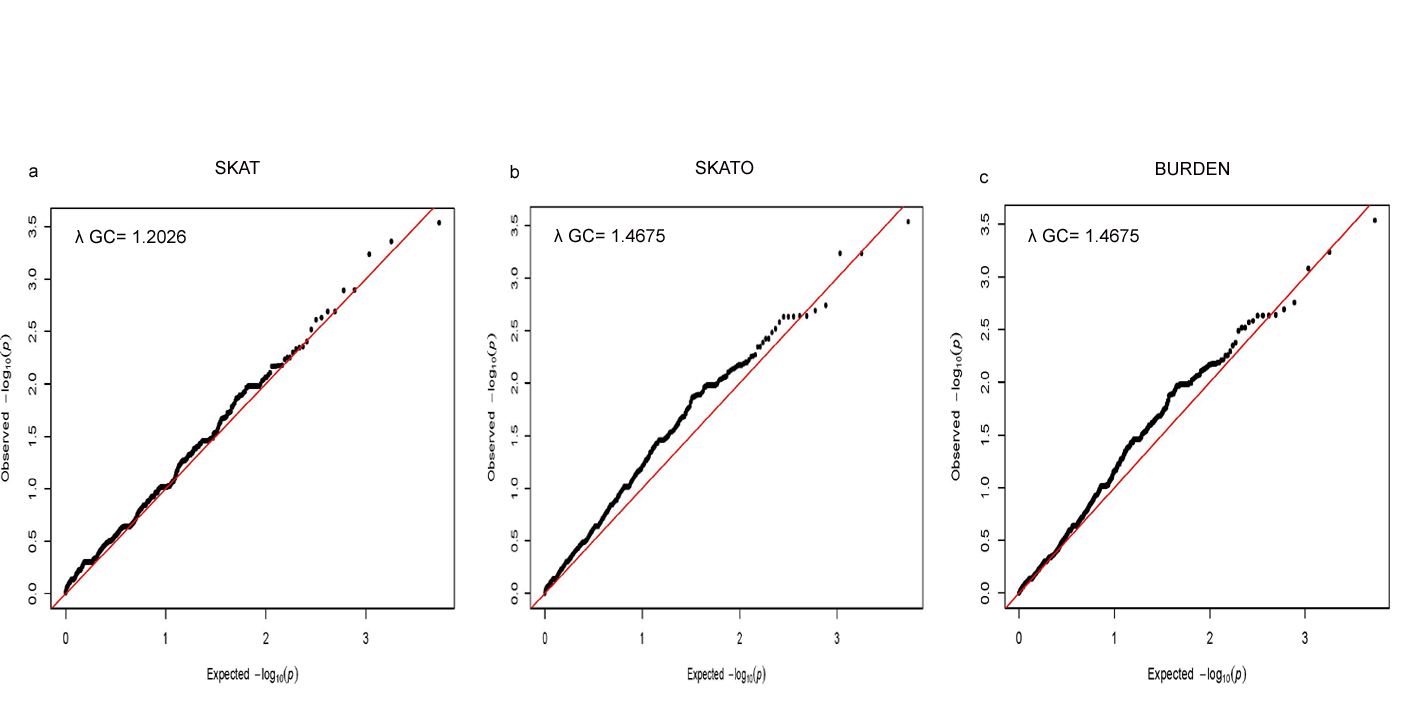

Supplement: Supplementary Figure 3 — Quantile-quantile plots of P-values. The observed negative logarithms of the P-values obtained using three types of gene-based methods for the two breeds are plotted against their expected values under the null hypothesis of no association. [file Figure_3.JPEG]

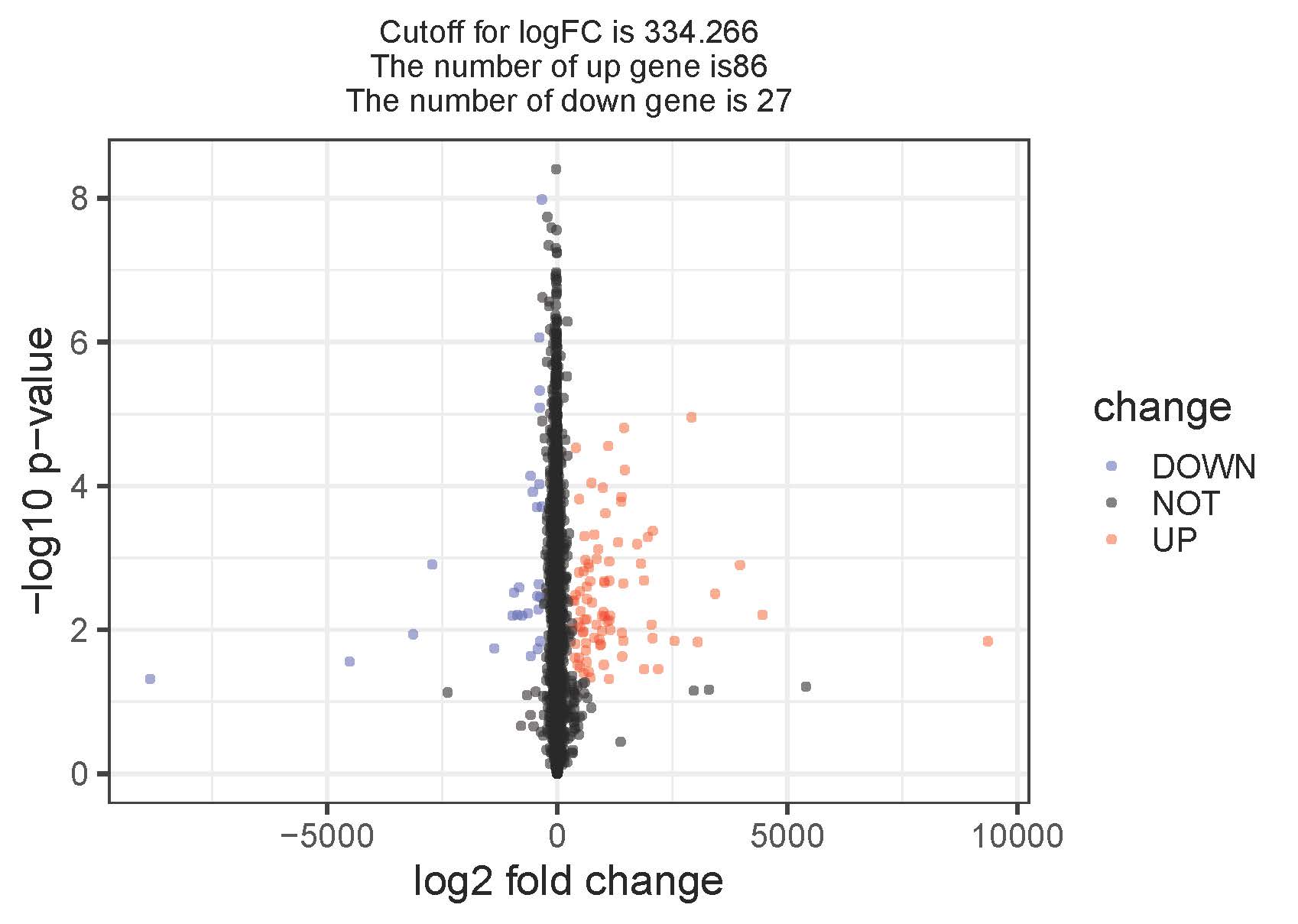

Supplement: Supplementary Figure 4 — Volcano plot of DEGs for the two cattle breeds. DEG expression profiling [with transcripts per kilo-base of exon model per million mapped reads (TPM) values] was constructed for the two breeds. Red plots represent genes that are significantly upregulated, and green plots represent genes that are significantly downregulated. [file Figure_4.JPEG]
